# Bactericidal Activity of the Bacterial ATP Synthase Inhibitor Tomatidine and the Combination of Tomatidine and Aminoglycoside Against Persistent and Virulent Forms of *Staphylococcus aureus*

**DOI:** 10.3389/fmicb.2020.00805

**Published:** 2020-05-05

**Authors:** Jean-Philippe Langlois, Guillaume Millette, Isabelle Guay, Alexis Dubé-Duquette, Suzanne Chamberland, Pierre-Étienne Jacques, Sébastien Rodrigue, Kamal Bouarab, Éric Marsault, François Malouin

**Affiliations:** ^1^Département de Biologie, Faculté des Sciences, Université de Sherbrooke, Sherbrooke, QC, Canada; ^2^Département de Pharmacologie et Physiologie, Faculté de Médecine et des Sciences de la Santé, Université de Sherbrooke, Sherbrooke, QC, Canada

**Keywords:** *Staphylococcus aureus*, small-colony variant, ATP synthase inhibitor, tomatidine, aminoglycoside, mode of action, membrane potential, reactive oxygen species

## Abstract

Tomatidine (TO), a steroid alkaloid, exerts a strong bactericidal activity on the infection-persistent phenotype of *Staphylococcus aureus*, the small-colony variant (SCV), with a minimal inhibitory concentration (MIC) of 0.06 μg/ml. Also, the combination of TO to an aminoglycoside (AMG) shows a strong synergistic effect against prototypical (WT) *S. aureus* (MIC 0.06 μg/ml), which is otherwise unaffected by TO alone (MIC > 128 μg/ml). We have recently established that the ATP synthase (subunit AtpE) was the molecular target of TO and that TO reduces the production of ATP in *S. aureus*. The purpose of this study was to understand how TO and the TO-AMG combination exert bactericidal activities against *S. aureus* SCV and WT strains, respectively. The impact of TO and of the TO-gentamicin (GEN) combination on the membrane potential and generation of reactive oxygen species (ROS) were determined using florescent probes. GEN uptake in WT was assessed in the presence of TO. Virulence of SCV and WT strains as well as of *in vitro*-selected mutants showing resistance to TO or the TO-GEN combination was evaluated in a murine thigh infection model. TO causes a reduction in membrane potential in both WT and SCV, but significant amounts of ROS are only produced in SCVs. Besides, the presence of TO improves the uptake of GEN by the WT strain and the combination TO-GEN generated 2.5-folds more ROS in WT, compared to that induced by GEN alone. Under anaerobic conditions, WT adopts a fermentative slow-growth phenotype and becomes susceptible to TO even if used alone. *In vivo*, TO- or TO-GEN-resistant strains were significantly altered in their ability to colonize tissues. These results shed light on the mechanism of action of TO and its synergy with AMGs against *S. aureus* WT. TO bactericidal activity against SCVs is attributable to both a critical drop in the membrane potential accompanied by a substantial ROS production. In the WT, TO helps GEN uptake and ROS is also important for the synergy. Acquiring resistance to TO significantly impairs virulence. The residual ATP synthase activity of SCVs might represent the Achilles’ heel of persistent *S. aureus*.

## Introduction

The world health organization (WHO) identified antibiotic resistance as a threat to humanity. Thus, developing new classes of antibiotics is imperative. Among the bacteria which represent the highest risk is *Staphylococcus aureus* (World Health Organization World Health Organization [WHO], 2017). *S. aureus* is overall the most prevalent pathogen in cystic fibrosis patients (Cystic Fibrosis Canada, 2018; Cystic Fibrosis Foundation, 2018). This pathogen can adapt to its environment, resist antibiotic treatments, evade the immune system and form biofilms (Liu, 2009; Le and Otto, 2015). The emergence of methicillin-resistant *S. aureus* (MRSA), which is often resistant to multiple classes of antibiotics, renders treatment even more complicated (Otto, 2012; Foster, 2017; Lakhundi and Zhang, 2018). Furthermore, in addition to the prototypical wild-type (WT) phenotype of *S. aureus*, the small-colony variant (SCV) is an alternate phenotype adopted by *S. aureus* which trades virulence for persistence traits (Tuchscherr et al., 2011; Suwantarat et al., 2018). SCVs most often have an impaired electron transport chain, which reduces ATP production and growth rate, very similarly to prototypical strains grown anaerobically (Proctor, 2019). SCVs are thus generally more resistant to aminoglycoside (AMG) antibiotics that need the membrane potential generated by the electron transport chain to cross the cytoplasmic membrane. Both the SCV and prototypical phenotypes are observed in the lungs of cystic fibrosis patients chronically infected with *S. aureus* (Proctor et al., 2006; Kahl et al., 2016; Cao et al., 2017).

Tomatidine (TO) is the aglycone precursor of α-tomatine, an antifungal compound naturally synthesized by solonaceous plants (Sandrock and Vanetten, 1998). We previously demonstrated that TO is a potent bacterial ATP synthase subunit c inhibitor that displays a strong activity against SCVs and targets very selectively the *Bacillales* (*Staphylococcus*, *Listeria*, *Bacillus*) (Lamontagne Boulet et al., 2018). Also, the combination of TO with an AMG, was shown to be synergistic and reduces the minimal inhibitory concentration of that class of antibiotics by up to 16-fold against prototypic *S. aureus* (WT), which is otherwise unaffected by TO alone (MIC of > 128 μg/mL) (Mitchell et al., 2012; Guay et al., 2018). Similarly, bedaquiline, also targeting the ATP synthase subunit c, is specifically active against *Mycobacterium tuberculosis* and other mycobacterial species (Hurdle et al., 2011). Targeting the energy production or membrane potential of bacteria has indeed been considered for the treatment of persistent infections in general (Hurdle et al., 2011; Nøhr-Meldgaard et al., 2018).

The stimulation of the production of reactive oxygen species (ROS) may be a secondary mechanism of action common to all bactericidal antibiotics (Kohanski et al., 2007). Antibiotic can stimulate the production of ROS, and more specifically, hydrogen peroxide (H_2_O_2_), superoxide (O_2_^–^•), and hydroxyl radicals (OH•). While the first two can be detoxified by bacteria with catalase and superoxide dismutase, respectively, bacteria are generally unable to detoxify the hydroxyl radicals (Kohanski et al., 2007; Van Acker et al., 2016). ROS are normal by-products of the aerobic respiration and are generated mainly by the oxidation of NADH by the electron transport chain (Vinogradov and Grivennikova, 2016). The overproduction of ROS under antibiotic pressure depletes NADH from the cells and creates a generalized oxidative stress on the bacteria. To confirm the hypothesis that TO bactericidal activity is linked to ROS production, it is essential to measure ROS production in the presence of TO and also to measure the activity of TO in the presence of an antioxidant or in the absence of oxygen. For example, the bactericidal activity of ciprofloxacin, a fluoroquinolone targeting bacterial topoisomerases, is decreased in the absence of oxygen (Van Acker and Coenye, 2017). Finally, to understand how TO increases AMG activity, the uptake of the latter by bacteria must be studied as AMGs enter bacteria more easily if the membrane integrity is compromised (Taber et al., 1987).

We report here how TO exerts its bactericidal activity, with or without the presence of an AMG, against both the WT and SCV phenotypes of *S. aureus*.

## Materials and Methods

### Strains and Growth Conditions

The WT strains used in this study were *S. aureus* Newbould (ATCC 29740) and *S. aureus* ATCC 29213. Strains NewbouldΔ*hemB* and ATCC 29213Δ*hemB* were used as SCV counterparts. These stable SCV strains were constructed by disrupting the *hemB* gene involved in the biosynthesis of heme by homologous recombination using the *ermA* cassette in both strains Newbould (Brouillette et al., 2004) and ATCC 29213 (Côté-Gravel et al., 2016). TO-resistant Δ*hemB* strains previously described by Lamontagne Boulet et al. (2018) and obtained by serial passage on sub-MICs of TO are also used in this study (Table 1). All strains were grown in tryptic soy broth (TSB) or on tryptic soy agar (TSA) at 35°C. When specified, oxygen did not exceed 10 ppm and hydrogen was less than 4% during anaerobic growth in an anaerobic chamber.

**TABLE 1:** Strains used in this study and their antibiotic susceptibility profiles.

*S. aureus* strain	Mutation	Growth phenotype	Microdilution MIC (μg/ml)^a^						TO inhibition zone (mm)^b^	
			TO	TO-Cys	GEN	TO-GEN	CIP	CIP-Cys	Aero	Anaero
Newbould (ATCC 29740)	–	Prototypic	>128	>128	0.25–1	0.06–0.12	0.12–0.25	0.5	0	23
NewbouldΔ*hemB*^c^	*hemB*:e*rmA*	SCV	0.06	0.25	8	–	0.12	0.25	25	28
NewbouldΔ *hemB_ccpA* (P07intR-1)^d^	*ccpA* G149V	SCV	0.25	0.5	8	–	0.12	0.25	–	–
NewbouldΔ *hemB_ccpA_atpE* (SaR1–1)^d^	*ccpA* G149V *aptE* A17S	SCV	>128	>128	8	–	0.12	0.25	–	–
ATCC 29213	–	Prototypic	>128	>128	0.25–1	0.06–0.12	0.25	0.5	–	–
ATCC 29213Δ *hemB*^e^	*hemB*:e*rmA*	SCV	0.06	0.25	8	–	0.25	0.5	–	–
ATCC 29213_*ndh2* (isolate #31)^f^	*ndh2* del G37	Prototypic	>128	>128	2	0.5–1	0.12–0.25	0.5	–	–
ATCC 29213_*ndh2*_*qoxC* (isolate #36)^f^	*ndh2* del G37 *qoxC* H140N	Prototypic	>128	>128	2–4	2–4	0.25	0.5	–	–

*^a^TO, tomatidine; Cys, cysteine 20 mM; GEN, gentamicin; CIP, ciprofloxacin. For the combination TO-GEN, TO was used at a concentration of 8 μg/ml. ^b^TO disk diffusion inhibition zone. Aero: under aerobic condition; Anaero: under anaerobic condition. ^c^Brouillette et al., 2004. ^d^Lamontagne Boulet et al., 2018. ^e^Côté-Gravel et al., 2016. ^f^Mutant obtained by serial passage on the TO-GEN combination in the present study. The isolate number is traceable in Supplementary Figure S1 and in the sequencing data (Supplementary Table S1). Gene annotations are from strain Newman (ndh2, NWMN_0811; qoxC, NWMN_0928).*

### Generation of *S. aureus* WT Mutants Resistant to the TO-Gentamicin (TO-GEN) Combination

Mutants resistant to the TO-GEN combination were generated by serial passage of *S. aureus* ATCC 29213 (30 passages of 48 h each) in a series of 2-fold dilutions of GEN (range, 0.06–64 μg/ml) in the presence of a fixed concentration of TO (8 μg/ml) in brain heart infusion (BHI) broth in 96-well plates. At each passage, the MIC was determined as the minimal concentration of the antibiotic combination inhibiting visible growth, and the well representing 0.5 MIC was diluted in fresh broth and used to inoculate (∼10^6^ CFU/ml) a new series of TO (8 μg/ml)-GEN dilutions for the next passage. At each passage, an aliquot of the 0.5 MIC well was also spread on BHI agar (BHIA), and the next day, three individual colonies were collected and frozen. Similar passages and isolate selection were also performed in parallel using GEN alone as the selective pressure. The whole genome of isolates showing an increased resistance to the TO-GEN combination or to GEN alone (three per passage of interest) was sequenced as described below.

### Whole-Genome Sequencing, Assembly, and Annotation

Whole-genome shotgun libraries were prepared and sequenced using Illumina technology. Briefly, genomic DNA was extracted using a Qiagen QIAamp DNA minikit and fragmented to a size of ∼200 to 400 bp by treatment with the double-stranded DNA (dsDNA) Shearase enzyme (Zymo Research) according to the manufacturer’s instructions. Libraries were then prepared according to Baby et al. (2018). Illumina sequencing was performed on an Illumina HiSeq 2000 sequencing system at the Plateau de Biologie Moléculaire et Génomique Fonctionnelle of the Institut de Recherche Cliniques de Montréal (Montreal, QC, Canada). Samples were multiplexed in a single sequencing lane, and approximately 1–2 million paired-end reads of 50 bp were obtained for each library. The resulting reads were *de novo* assembled using a Roche gsAssembler, version 2.6. Contig sequences were aligned and annotated using the reference genome of *S. aureus* strain Newman with ABACAS (Assefa et al., 2009) and RATT (Otto et al., 2011). Four independently sequenced *S. aureus* ATCC 29213 genomes were first compared to the reference *S. aureus* Newman genome to identify and eliminate inherent single nucleotide variants (SNVs) from the subsequent comparison of *S. aureus* ATCC 29213 to resistant isolates. TO-GEN resistant *S. aureus* ATCC 29123 sequences were then compared against the parental strain *S. aureus* ATCC 29213 to identify SNVs or indels uniquely associated with the resistance to the drug combination and that were thus not present in the isolates selected on GEN alone. Only SNVs or indels present in each of the three isolates selected at a given passage and subsequently retained in the genomes of isolates collected in all subsequent passages were considered. The accession number for all the sequenced strains is: PRJNA609713.

### Antibiotic Susceptibility Testing

Antibiotic MICs were determined by a broth microdilution technique according to the recommendations of the Clinical and Laboratory Standards Institute [CLSI], 2018, except that the medium used was TSB instead of Mueller Hinton broth to better sustain the growth of SCV strains. Microtiter plates were incubated for 20–24 h or 48 h for the prototypic and SCV phenotypes, respectively, under aerobic conditions at 35°C. TO susceptibility was also determined using a disc diffusion method under aerobic and anaerobic conditions. Four-mm discs containing 50 μg of TO were placed on pre-inoculated TSA plates and inhibition zones were measured after 48 h. During anaerobic growth in the anaerobic chamber, oxygen did not exceed 10 ppm and hydrogen was under 4%.

### Cell Surface Hydrophobicity

This assay was performed according to that described by Lye et al. (2010) with a few modifications. Briefly, bacteria were incubated 18 h in TSB, washed and suspended in PBS. The A_600__*nm*_ was adjusted to 1.0 (A_0_) in PBS, mixed with xylene, vortexed 1 min and let to rest 30 min to allow the separation of the two phases. The xylene to PBS ratio was 1:9 for *S. aureus* Newbould and derivatives, and 1:8 for *S. aureus* ATCC 29213 and derivatives. The aqueous (bottom) phase was recovered and the A_600__*nm*_ was again measured (A). The hydrophobicity percentage was calculated as follow:

(1) $$\frac{A_{0}-A}{A_{0}}\times 100.$$

### Membrane Potential Measurement

The relative membrane potential was estimated using the fluorescent dye indicator 3,3’-diethyloxacarbocyanine iodide (DiOC_2_) (ThermoFisher), according to the manufacturer’s protocol. Briefly bacteria were incubated 18 h on TSA plates containing various concentrations of TO. Alternatively, bacteria were incubated in TSB for 1 h to test the effect of the TO-GEN combination on the membrane potential. Bacterial cells were then harvested, washed and suspended in PBS. DiOC_2_ (3 mM) was added and incubated 30 min at 35°C. Using a FACScalibur with a 488 nm laser, cells were analyzed with the red (FL3-H) and green (FL1-H) channels. A higher red on green ratio (MFI) represents a higher membrane potential. Data were analyzed using the FCS Express program. Results are expressed relatively to the MFI determined for *S. aureus* in the absence of antibiotic (representing a MFI of 100%).

### Reactive Oxygen Species (ROS) Quantification

Bacteria were incubated overnight on TSA. A few colonies were suspended in 10 ml TSB and incubated 2 h. Afterward, A_600__*nm*_ was adjusted to 0.1, 10 μM 2’,7’-dichlorodihydrofluorescein diacetate (H_2_DCFDA) was added and incubated 1 h. Bacteria were then washed and suspended in fresh TSB. Finally, bacteria were added to a black, flat-bottom 96-well plate, in triplicate. Strains were exposed to 4 × MIC, or 8 μg/ml if bacteria were inherently not susceptible to the drug (e.g., prototypic *S. aureus* vs TO). Using a TECAN Genios Fluorescence plate reader, the plate was read using the fluorescein settings. Each well was read at 9 positions per well every 15 min, over a period of 13 h under aerobic conditions. The highest fluorescence value (peak) was considered and background fluorescence was subtracted. Data are expressed as a relative fluorescence intensity (RFI). The control condition without antibiotic represented a RFI of 1.0 (Van Acker et al., 2016).

### GEN Uptake Assay

The aminoglycoside uptake assay was done according to Loughman et al. (2016). GEN was tagged using the Texas Red-X succinimidyl ester (ThermoFisher) at a molar ratio of 300:1 and incubated overnight at 4°C with agitation. To remove unbound Texas Red, the dye remover column (Thermo Fisher) was used according to the manufacturer’s protocol. Bacteria were adjusted to an A_600__*nm*_ of 0.1 and incubated 2 h at 35°C with agitation in the presence of 128 μg/ml of the Texas Red-GEN conjugate. When TO was added during the uptake assay, it was used at 8 μg/ml. Plates were read with a TECAN Genios Fluorescence plate reader with the 535/595 nm filters.

### Kill Kinetics

Kill kinetics were performed under aerobic and anaerobic conditions. Bacteria were inoculated at ∼10^5^–10^6^ CFU/ml in TSB, with or without antibiotics, and grown at 35°C with agitation. At several time points, bacteria were sampled, serially diluted and 10 μl were plated on TSA for CFU count.

### Mouse Thigh Infection Model

A previously described mouse thigh infection model (Gudmundsson and Erlendsdóttir, 1999) was used to investigate the extent of colonization by *S. aureus* and TO- and TO-GEN-resistant mutants *in vivo*. Female CD-1 mice were rendered neutropenic by intraperitoneal delivery of 150 and 100 mg/kg of cyclophosphamide at day -4 and -1, respectively. On the day of the infection, bacteria grown on TSA were suspended at 1 × 10^6^ CFU/ml in PBS. Using a 1-ml syringe with a 27G needle, 0.1 ml of the bacterial suspension (1 × 10^5^ CFU) was injected into both thighs of each mouse. The animals were euthanized 8 h post-infection, and the thigh muscles were harvested and homogenized in 3 ml of PBS using a Kinematica Polytron homogenizer 10–35 GT (Kinematica, Bohemia, NY, United States). CFU were determined following serial dilution of homogenates in PBS and plating on TSA. The plates were incubated 24 h at 35°C for ATCC 29213 and the TO-GEN combination-resistant mutant (ATCC 29213_*ndh2*_*qoxC*), and 48 h for the parental SCV (NewbouldΔ*hemB*) and TO-resistant SCV mutants (*ccpA* and *atpE*).

### Statistical Analyses

Statistical analyses were carried out with the GraphPad Prism Software (v.8.00). Statistical tests used for each experiment are specified in the legends of associated figures.

## Results

### Antibiotic Susceptibility Profiles and Selection of TO-GEN Resistant Strains

The antibiotic susceptibility profiles of the strains and mutants used in this study are reported in Table 1. As expected, prototypic *S. aureus* (Newbould or ATCC 29213) are inherently non-susceptible to TO (MIC > 128 μg/ml) and susceptible to aminoglycosides (here GEN). Conversely, the SCV phenotype is hypersusceptible to TO (MIC 0.06 μg/ml) but more resistant to GEN (MIC 8 μg/ml) due to its defect in the *hemB* gene and defective electron transport chain. Besides, the mutants derived from NewbouldΔ*hemB* show increasing resistance to TO after serial passage as previously reported by Lamontagne Boulet et al. (2018); a *ccpA* mutation (catabolite control protein A) was associated with an intermediate level of resistance (TO MIC of 0.25 μg/ml) and a full resistance to TO (MIC > 128 μg/ml) was associated to a mutation in the drug target, the ATP synthase subunit c (*atpE*). On the other hand, the present study shows that resistance of the prototypic strain *S. aureus* ATCC 29213 toward the TO-GEN combination only increased from 0.06–0.12 μg/ml to 2–4 μg/ml upon 30 passages in the presence of the combination (Supplementary Figure S1). Again, the higher level of resistance was achieved by a stepwise process; first a mutation in the *ndh2* gene (coding for a type-II NADH dehydrogenase) providing an intermediate level of resistance (GEN MIC of 0.5–1 μg/ml in the presence of 8 μg/ml TO) and a second mutation in *qoxC* (coding for a quinol oxidase, subunit 3) for the highest, although modest, level of achievable resistance (GEN MIC of 2–4 μg/ml in the presence of 8 μg/ml TO). Table 1 shows the isolate number of the sequenced strains that were retained for further investigation. The isolate numbers are traceable in Supplementary Figure S1 and in the sequencing data (Supplementary Table S1). Gene annotations are from strain Newman (*ndh2*, NWMN_0811; *qoxC*, NWMN_0928). The accession number for all the sequenced strains is: PRJNA609713. We also provide the information of interest in Supplementary Table S1 in which we highlighted the mutations that were considered for isolates 31 and 36, i.e., SNVs or indels uniquely associated with the resistance to the drug combination (TO-GEN) and that were thus not present in the isolates selected on GEN alone. Only SNVs or indels present in each of the three isolates selected at a given passage and subsequently retained in the genomes of isolates collected in all subsequent passages were considered. In these selected TO-GEN resistant mutants, the MIC of GEN alone also increased from 0.25–1 μg/ml to a maximum of 2–4 μg/ml, whereas when GEN is used alone in serial passage, the MIC of GEN can reach ≥8 μg/ml after 30 passages (data not shown).

### Strains Resistant to TO or to the TO-GEN Combination Exhibit a Modified Cell Surface Hydrophobicity

**FIGURE 1:**
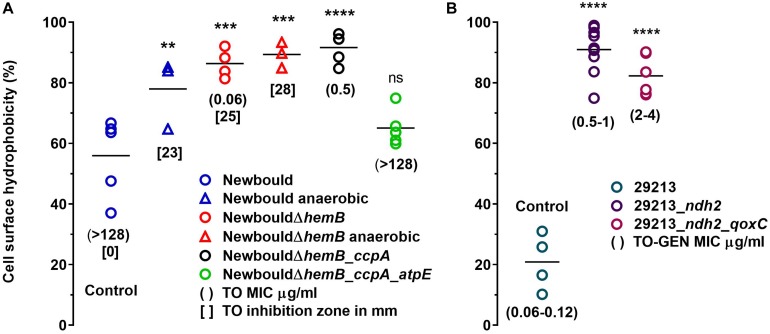
Cell surface hydrophobicity of *S. aureus* strains including those resistant to TO **(A)**, or resistant to the TO-GEN combination **(B)**. In **(A)**, surface hydrophobicity of Newbould and Newbould Δ*hemB* was also measured after growth in anaerobic conditions (triangles). Each strain was tested four to nine times independently and the medians are represented by the horizontal bars. Statistical analysis was performed using a one-way ANOVA with a Dunnett’s multiple comparison test, comparing each strains to *S. aureus* Newbould (Control in **A**) or *S. aureus* ATCC 29213 (Control in **B**); ****p < 0.0001, ***p < 0.001, **p < 0.01, and ns, not statistically significant.

**FIGURE 2:**
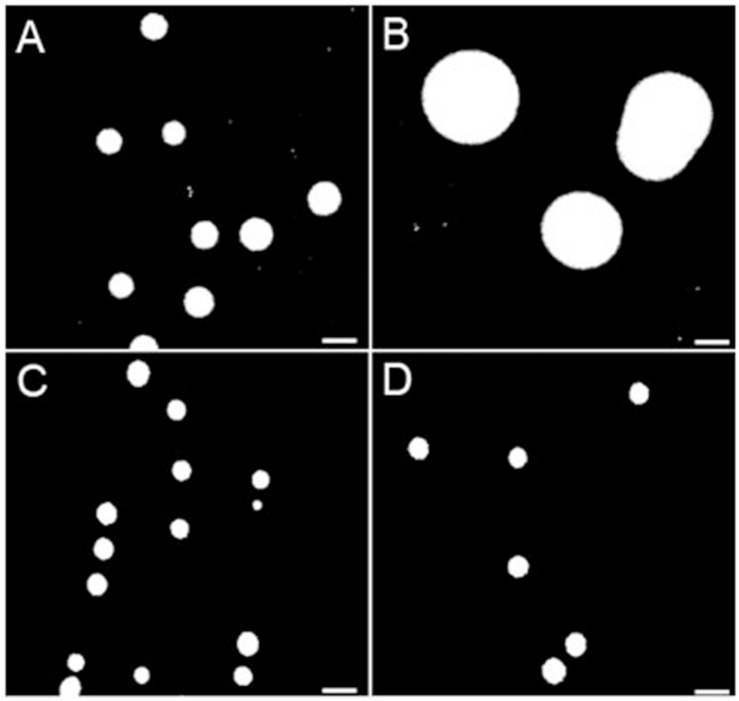
Colony sizes of *S. aureus* strains Newbould and NewbouldΔ*hemB* grown under aerobic and anaerobic conditions. **(A)** Grown aerobically, the average colony size of NewbouldΔ*hemB* was 0.86 and 2.26 mm for the WT strain Newbould **(B)**. In anaerobic conditions, both NewbouldΔ*hemB* **(C)** and Newbould **(D)** showed small colony sizes that were reduced to 0.64 mm.

Surface hydrophobicity patterns were found to be generally correlated with MICs (Figure 1). The prototypic *S. aureus* strain Newbould (inherently non-susceptible to TO) as well as the SCV mutant NewbouldΔ*hemB*_*ccpA*_*atpE* highly resistant to TO were significantly less hydrophobic than the TO-susceptible *S. aureus* NewbouldΔ*hemB* strain (Figure 1A). High and low cell surface hydrophobicity were characteristics that applied to other SCV and prototypical strains, respectively (Supplementary Figure S2). Also seen in Figure 1A, the mutation in *ccpA* conferring low-level resistance to TO did not influence cell surface hydrophobicity compared to the parental strain *S. aureus* NewbouldΔ*hemB*. Besides, hydrophobicity tests were also performed under anaerobic conditions (Figure 1A). Notably, in the absence of oxygen, the prototypical strain *S. aureus* Newbould adopts a small-colony phenotype (Figure 2), as for all prototypical strains grown anaerobically (Proctor, 2019), and becomes highly susceptible to TO (Table 1, TO inhibition zone of 23 mm) similarly to that seen in either aerobic or anaerobic conditions for the SCV strain NewbouldΔ*hemB* (Table 1, TO inhibition zone of 25 and 28 mm, respectively for the two growth conditions). Accordingly, susceptibility to TO is again correlated to a high cell surface hydrophobicity (Figure 1A). Interestingly, strains resistant to the TO-GEN combination demonstrated the opposite hydrophobicity pattern than that observed for the TO-resistant strains (Figure 1B). A mutation in *ndh2* in the prototypical strain ATCC 29213 shifted the cell surface hydrophobicity from low to high while the additional *qoxC* mutation did not contribute further to the already high cell surface hydrophobicity.

Hence, a low hydrophobicity is associated to a high-level of resistance to TO (MIC > 128 μg/ml), whereas a high hydrophobicity is associated with resistance to the TO-GEN combination albeit the latter is a relatively low-level of resistance (GEN MIC in combination 2–4 μg/ml). As such, since a high hydrophobicity is also associated to high TO susceptibility, it appears that exposure of bacteria to the TO-GEN combination prevents development of high-level resistance. Besides, cell surface hydrophobicity may be linked to surface charge or membrane energization and the membrane potential was thus studied next.

### Membrane Potential Is Reduced in Presence of TO

**FIGURE 3:**
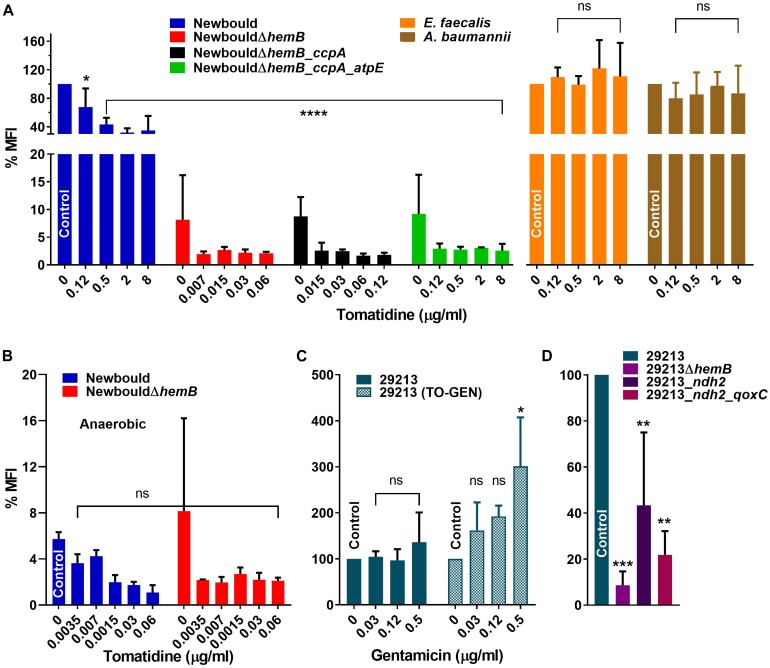
Membrane potential (MFI) of bacterial strains in the presence of TO or TO-GEN. **(A)** *S. aureus* Newbould, Newbould derivative mutants and non-target species in presence of TO. **(B)** *S. aureus* Newbould and Newbould Δ*hemB* in presence of TO under anaerobic conditions. The MFI was relative to that of strain Newbould grown aerobically without antibiotic as in **(A)**. **(C)** *S. aureus* ATCC 29213 in presence of GEN or of GEN in combination with TO (8 μg/ml). **(D)** *S. aureus* ATCC 29213 and derivative mutants. Statistical analyses were performed using a one-way ANOVA with a Dunnett’s multiple comparison test, comparing each panel to the indicated control; ****p < 0.0001, ***p < 0.001, **p < 0.01 and *p < 0.05.

TO targets the ATP synthase subunit c and we therefore investigated its effect on membrane potential. As expected, the first observation was that the deletion of *hemB* in strain Newbould and the consequent impairment of its electron transport chain significantly reduced the membrane potential (Figure 3A, Newbould vs NewbouldΔ*hemB*). Furthermore, there was no difference in the membrane potential of *S. aureus* NewbouldΔ*hemB*, *S. aureus* NewbouldΔ*hemB*_*ccpA* and NewbouldΔ*hemB*_*ccpA*_*atpE*, which were all very low compared to that of the prototypical strain Newbould. Surprisingly, despite the very high MIC of TO for strain Newbould (MIC > 128 μg/ml), a dose-dependent reduction of membrane potential was observed in that strain when exposed to TO. We then tested additional strains for measurement of membrane potential, notably *S. aureus* Newman (a well-known reference strain), and strain SH1000 (a *rsbU*-corrected 8325-4 derivative which now produces large amounts of biofilm). We observed the same type of results as that seen with strain Newbould; the membrane potential decreases in presence of TO (Supplementary Figure S3). The highest TO concentration tested was 8 μg/ml and even at this relatively low concentration compared to the MIC, the membrane potential of strain Newbould was reduced by more than 65% (Figure 3A). The reduction of the membrane potential observed with *S. aureus* Newbould seems to stabilize at around 35% of that of Newbould in the absence of TO. Since *S. aureus* Newbould had a reduced membrane potential in the presence of TO, the specificity of the assay was tested with Gram-positive and Gram-negative bacterial species that were not part of the *Bacillales* (TO MIC > 128 μg/ml), and for which the ATP synthase subunit c amino acid sequence diverge. Both *Enterococcus faecalis* and *Acinetobater baumannii* membrane potentials were not affected by TO (Figure 3A), confirming that the observed effects of TO were specific to *S. aureus*.

When TO was added to SCV strains, the already low membrane potential was reduced further compared to the no antibiotic control for both *S. aureus* NewbouldΔ*hemB*, NewbouldΔ*hemB*_*ccpA* and NewbouldΔ*hemB*_*ccpA*_*atpE* although the reductions were not statistically significant (Figure 3A). For NewbouldΔ*hemB* and NewbouldΔ*hemB*_*ccpA*, no growth was possible above 0.12 μg/ml of TO, whereas the membrane potential of the *atpE* mutant did not fell below 2% of that of strain Newbould even in the presence of 8 μg/ml of TO (Figure 3A).

Membrane potential was also measured after anaerobic growth. As mentioned above, WT Newbould adopts a fermentative slow-growth phenotype in absence of oxygen (Figure 2) and becomes susceptible to TO (Table 1). Under such anaerobic conditions, the membrane potential of *S. aureus* Newbould was as low as that observed for *S. aureus* NewbouldΔ*hemB* (Figure 3B). A reduction in membrane potential was further observed for both strains in presence of very low TO concentrations and no growth was possible above 0.12 μg/ml of TO. These results suggest that at a relatively low concentration, TO affects susceptible SCV strains or anaerobically grown WT *S. aureus* to a point where the membrane potential falls below a critical level (<2%). The loss of respiration in SCVs that is due to a mutation (i.e., *hemB* in this case) makes them susceptible to TO in presence or absence of oxygen, whereas prototypical strains are only susceptible to TO in anaerobic conditions (Table 1) and revert to a non-susceptible phenotype when respiration starts again in the presence of oxygen.

### TO Influences the Membrane Potential When Combined With GEN

Since we observed that TO reduces the membrane potential of *S. aureus* (Figure 3A), it was hypothesized that the combination with GEN could modulate this effect. First, when measuring the effect of GEN on the membrane potential, no difference or modulation of the membrane potential was observed with increasing concentrations (≤MIC) of GEN (Figure 3C, left panel). Addition of TO to the same set of GEN concentrations caused of significant increase in membrane potential (Figure 3C, right panel). Besides, the membrane potential of TO-GEN resistant mutants (*ndh2* or *ndh2_qoxC* mutants) was significantly lower than that of the parental strain ATCC 29213 (Figure 3D). Since GEN entry into cells depends on a high membrane potential (Mates et al., 1982), observations in Figures 3C,D could account on one hand for the increase susceptibility of ATCC 29213 to GEN when TO is present and on the other hand, the decreased susceptibility of the TO-GEN resistant mutants to either GEN alone or the TO-GEN combination. Finally, the membrane potential of either the *ndh2* or *ndh2_qoxC* mutant was still higher than that of the ATCC 29213Δ*hemB* mutant (Figure 3D), which could explain why the former mutants are not susceptible to TO compared to strains of the SCV phenotype (Table 1).

### ROS Are Produced in Susceptible Strains Exposed to Either TO Alone or to the TO-GEN Combination

**FIGURE 4:**
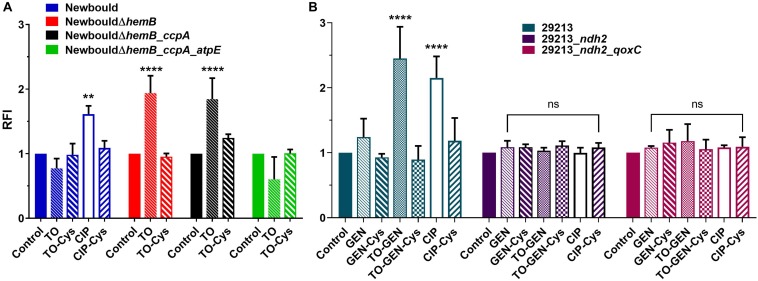
ROS production (RFI) in *S. aureus* Newbould and derivative mutants **(A)**, and in *S. aureus* ATCC 29213 and derivative mutants **(B)**. Strains were grown without antibiotic or in the presence of TO (4 × MIC for susceptible strains and 8 μg/ml for resistant strains), ciprofloxacin (CIP, at 4 × MIC), GEN (4 × MIC), or the combination of TO (8 μg/ml) and GEN (4 × MIC). The reducing agent cysteine (Cys, 20 mM) was added where specified. Statistical analyses were performed using a two-way ANOVA with a Dunnett’s multiple comparison test, comparing each group to their control without antibiotic; ****p < 0.0001 and **p < 0.01.

ROS production by the TO-susceptible and TO-intermediately resistant SCV strains NewbouldΔ*hemB* and NewbouldΔ*hemB*_*ccpA*, respectively, was significantly induced in presence of TO at 4 × MIC (Figure 4A). Also, as expected, the ROS production induced by TO was attenuated by the antioxidant effect of cysteine. On the opposite, the intrinsically TO-resistant WT *S. aureus* strain Newbould or the TO-resistant SCV strain NewbouldΔ*hemB_ccpA_atpE* did not produce more ROS in the presence of TO at 8 μg/ml than that observed without antibiotic (Figure 4A). Ciprofloxacin (4 × MIC) used as a positive control did provoke ROS production in strain Newbould, an effect also reverted by cysteine. Therefore, TO only induces ROS production in TO-susceptible or TO-intermediately resistant strains. These results were obtained under aerobic conditions.

When TO at 8 μg/ml was added to GEN at 4 × MIC, *S. aureus* ATCC 29213 demonstrated a significant increase in ROS production compared to that induced by GEN alone (Figure 4B). ROS production caused by GEN alone did not differ from that of the no antibiotic control. In the TO-GEN resistant strains, the *ndh2* mutation (either alone or combined with the *qoxC* mutation) completely suppressed the ROS production induced by the presence of TO. In conclusion, in the *S. aureus* WT background, ROS are only produced in susceptible strains when TO is added to GEN. This could be another contributor to the TO-GEN synergy. A mutation in *ndh2* protects bacteria against the TO-induced ROS production.

### Effect of TO on GEN Uptake

**FIGURE 5:**
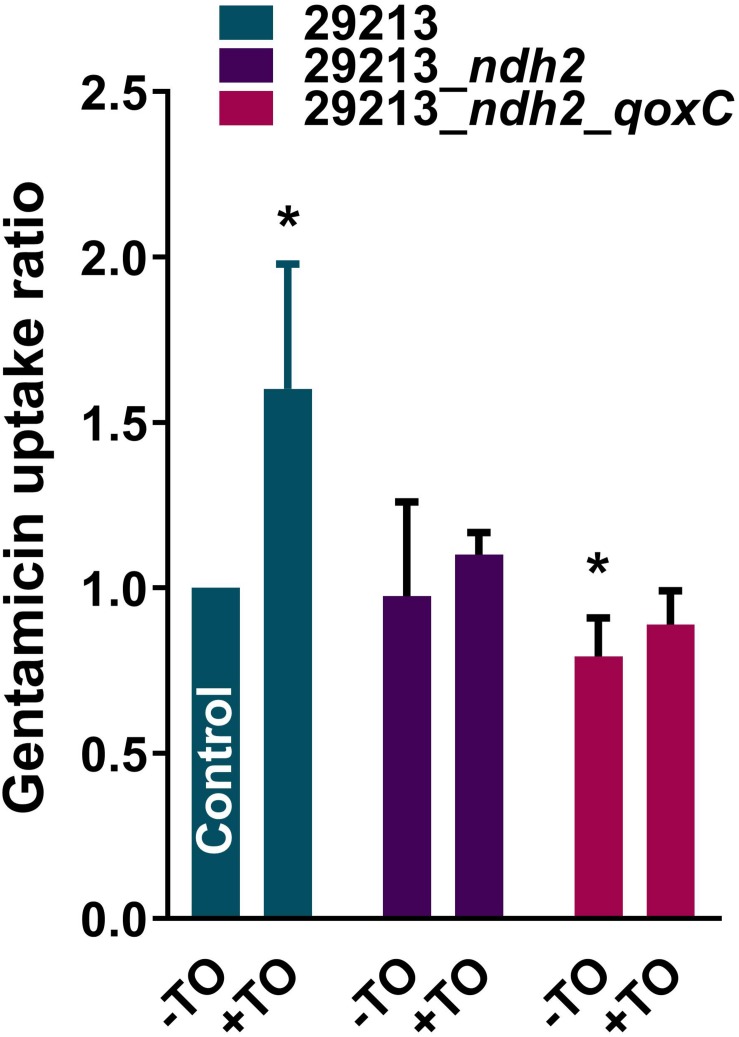
GEN uptake by *S. aureus* strains. GEN uptake was measured over a 2 h-period for *S. aureus* ATCC 29213 and mutants resistant to the TO-GEN combination in the absence (-TO) or presence (+TO) of TO at 8 μg/ml. Data are expressed relatively to the uptake of GEN by the WT strain ATCC 29213 in the absence of TO (Control). Statistical analysis was performed using a one-way ANOVA with a Dunnett’s multiple comparison test, using ATCC 29213 in the absence of TO as the control; *p < 0.05.

The uptake of GEN by *S. aureus* was measured to determine if TO modulates GEN uptake when both are combined and also to assess the impact of the *ndh2* and *qoxC* mutations (Figure 5). There was a significant increase in the uptake of GEN (∼1.6×) by the WT strain ATCC 29213 in the presence of TO (8 μg/ml). This was consistent with the increased membrane potential seen when TO and GEN are combined (Figure 3C). There was no difference in GEN uptake between *S. aureus* ATCC 29213 and the *ndh2* mutant but there was a significant reduction of the uptake of GEN in the double mutant *ndh2*_*qoxC*. Also, for the *ndh2* and *ndh2*_*qoxC* mutants, addition of TO did not increase the uptake of GEN as seen in the WT strain. Therefore, resistance to the TO-GEN combination is associated with a reduction in GEN uptake.

### Activity of TO and the TO-GEN Combination Under Aerobic and Anaerobic Conditions

**FIGURE 6:**
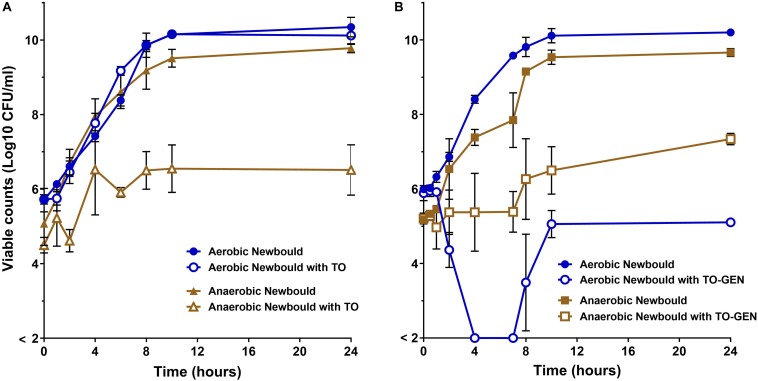
WT *S. aureus* Newbould kill kinetics by antibiotics. **(A)** *S. aureus* Newbould in the presence of TO (16 μg/ml) alone or **(B)** in the presence of the combination of TO (16 μg/ml) and GEN at 1 μg/ml (TO-GEN). Kill kinetics were determined in aerobic and anaerobic conditions as indicated. The log_10_ CFU/ml indicated at each time point represent the average of three independent experiments. The detection threshold was 2 log_10_ CFU/ml.

**FIGURE 7:**
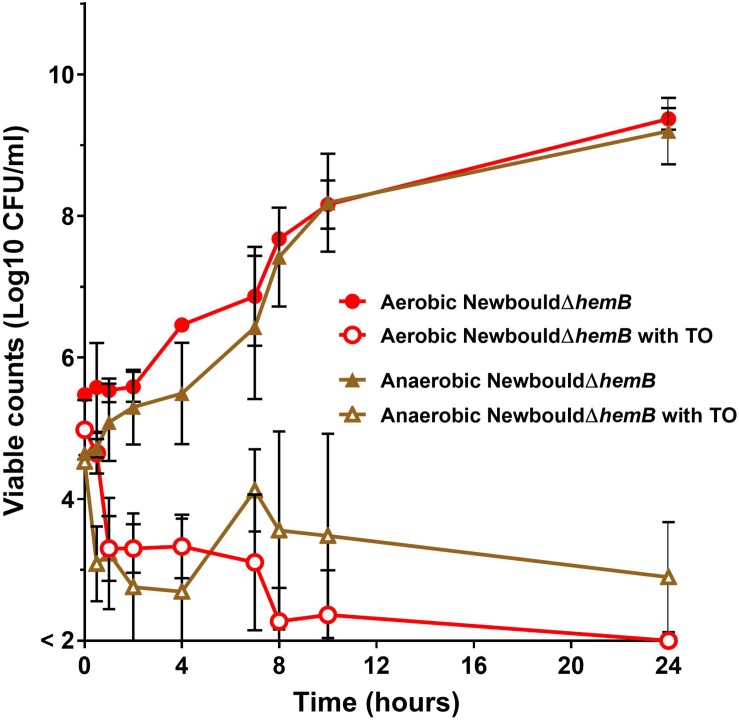
*S. aureus* SCV kill kinetics by antibiotics. *S. aureus* NewbouldΔ*hemB* in the presence of TO (16 μg/ml). Kill kinetics were determined in aerobic and anaerobic conditions as indicated. The log_10_ CFU/ml indicated at each time point represent the average of three independent experiments. The detection threshold was 2 log_10_ CFU/ml.

Time-kill experiments for *S. aureus* strain Newbould (Figure 6) and NewbouldΔ*hemB* (Figure 7) were performed for a period of 24 h under aerobic and anaerobic conditions. First, strain Newbould, like all prototypical strains, is not susceptible to TO in aerobic conditions but displays a slow fermentative growth in the absence of oxygen (Figure 2), and thus becomes inhibited by TO in anaerobic conditions (Table 1). Figure 6A shows that, while this growth inhibition is bacteriostatic and not cidal, Newbould is indeed only inhibited by TO in anaerobic conditions. The switch from the normal phenotype (respiration) to the slow-growth phenotype (fermentative growth) makes impossible to evaluate the contribution of oxygen to the mechanism of action of TO against strain Newbould under these two growth conditions. However, a direct comparison of aerobic and anaerobic conditions is possible in Figures 6B where the combination TO-GEN is used against Newbould. Figure 6B clearly shows that more killing can be achieved in aerobic conditions for the TO-GEN combination against the WT strain. Besides, compared to that seen with WT (Figure 6A), TO used alone is able to kill the genetically stable SCV strain under both aerobic and anaerobic conditions (Figure 7).

### Fitness of TO and TO-GEN Resistant Mutants in a Thigh Infection Model

**FIGURE 8:**
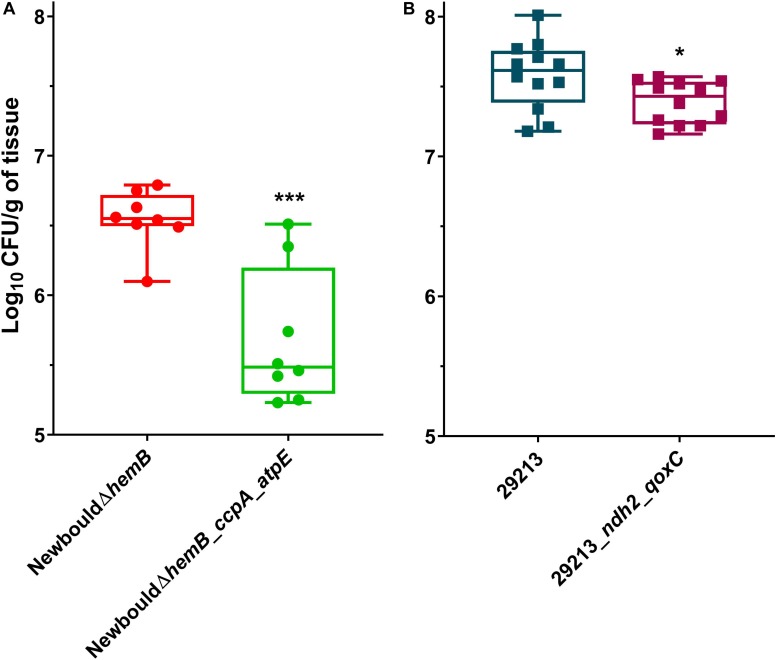
Mouse thigh infections by *S. aureus* strains. **(A)** NewbouldΔ*hemB* colonization was compared to that of NewbouldΔ*hemB*_*ccpA*_*atpE*. **(B)** ATCC 29213 colonization was compared to that of ATCC 29213_*ndh2*_*qoxC*. CFU were determined 8 h post-infection. Each symbol represents one thigh tissue. The median for each group is indicated by the horizontal bar. Significance between the median log_10_ CFU per gram of tissue in each panel was determined using an unpaired *t*-test two tailed; ***p < 0.001, *p < 0.05.

Since resistance to TO and the TO-GEN combination was achieved in *S. aureus* by various mutations in the ATP synthase or electron transport chain, we hypothesized that these mutations would affect fitness *in vivo*. Indeed, we observed a growth deficit of over 1 log_10_ of CFU for the TO-resistant strain NewbouldΔ*hemB*_*ccpA*_*atpE* compared to the parental SCV strain NewbouldΔ*hemB* in the thigh infection model (Figure 8A). Although not as much affected, colonization of the TO-GEN resistant mutant ATCC 29213_*ndh2*_*qoxC* was also significantly altered when compared to its prototypic parental strain ATCC 29213 (Figure 8B). The colonization of the TO-intermediate resistant mutant NewbouldΔ*hemB_ccpA* was not significantly reduced compared to its parental strain (Supplementary Figure S4), although a trend could be observed. Taken together, these results show that *S. aureus* resistance to TO alone or in combination with GEN requires a trade-off that decreases the ability of resistant strains to infect *in vivo*.

## Discussion

TO is an efficient antibiotic against the SCV phenotype of *Bacillales* such as *S. aureus*, *L. monocytogenes* and *B. subtilis* (Guay et al., 2018). Although TO lacks activity against the WT phenotype, the combination with an aminoglycoside reduces GEN, tobramycin or kanamycin MICs by up to 16-fold (Mitchell et al., 2012). It was previously shown that TO targets the ATP synthase subunit c, reduces ATP production (Lamontagne Boulet et al., 2018), and could consequently reduce synthesis of macromolecules (Mitchell et al., 2011). As aminoglycosides lead to erroneous or aborted protein translation, both TO and GEN can thus reduce the ability of bacteria to thrive and maintain their cellular integrity.

Laboratory-selected TO-resistant *S. aureus* SCVs mutated in the *atpE* gene coding for the ATP synthase subunit c were shown to have a lower ATP production than the parental SCV strain NewbouldΔ*hemB*, which already produced much lower ATP amounts than the prototypical strain Newbould as evaluated in an inverted membrane vesicle ATP synthesis assay (Lamontagne Boulet et al., 2018). Gaddy et al. (2015), reported that a restriction in nutrients, and thus a lower energy production, can induce a modification in lipid composition and phosphorylation of the membrane. Cell surface hydrophobicity is dependent, among other factors, of membrane lipid composition and modification of membrane lipids with L-lysine (Peschel et al., 2001). *S. aureus* can also modify cell surface hydrophobicity by attaching teichoic acids and modifying D-alanylation levels in cell wall peptidoglycan (Keinhörster et al., 2019), and synthesis of teichoic acids is dependent of electron transport (Proctor, 2019). Since TO is a highly hydrophobic molecule, a less hydrophobic surface might limit the physical interactions between the antibiotic and bacteria. We showed here that *S. aureus* prototypic cells that produce large amounts of ATP and that display a high membrane potential and a low cell surface hydrophobicity are “naturally” resistant to TO. Whether or not the low surface hydrophobicity is a result of the membrane potential or the high energy production allowing adequate biogenesis of the cell membrane and teichoic acids is still unknown. On the other hand, *S. aureus* strains resistant to the TO-GEN combination had their surface hydrophobicity increased despite the presence of TO. In this case, the consequence of carrying a *ndh2* mutation for the bacteria would decrease the membrane potential and limit the interaction and uptake of the hydrophilic GEN at the expense of an increased affinity for TO. As such, only low-level resistance to the TO-GEN combination could be achieved through serial passage.

The first mutation selected in the prototypical strain ATCC 29213 and that resulted in resistance to the TO-GEN combination was indeed found in type-II NADH dehydrogenase (*ndh2* gene). In *S. aureus*, this protein is responsible for the oxidation of NADH in NAD with production of ROS as by-products. This protein is associated with a proton transporter, MpsABC (Mayer et al., 2015). Both proteins form the complex I of the electron transport chain. Inhibiting the dehydrogenase activity reduces the proton gradient and the generation of ROS (Kohanski et al., 2007; Mayer et al., 2015). The second mutation we found in mutants resistant to the TO-GEN combination is in the *qoxC* gene coding for the main terminal cytochrome *aa3* menaquinol oxidase at the end of the electron transport chain (Götz and Mayer, 2013; Proctor, 2019). Hence, mutations reducing the activity and the capability of the electron transport chain decrease GEN uptake (Figure 5).

Membrane potential generated by the electron transport chain is essential for the production of ATP and some aspects of virulence and this sequence of events represents an interesting and underexploited target for the discovery of new antibiotics (Proctor et al., 2002). For example, even *M. tuberculosis* persister cells need to maintain a membrane potential in order to survive (Moreira et al., 2016). This raises the question of whether it exists or not a minimal membrane potential threshold necessary for survival of bacteria. For *S. aureus*, we found that there was a critical membrane potential threshold correlating with cell viability (Figure 3).

Antibiotic-induced ROS production may represent the ultimate bactericidal step in antibiotic action, but this view is currently debated. The main argument against this point of view is the fact that a large number of bacteria are still killed by antibiotics under anaerobic conditions and some of the past studies were performed using the fluorescence probe hydroxyphenyl fluorescein (HPF), which tends to self-oxidize (Fang, 2013). On the other hand, advocates maintain that induction of ROS production is common to all bactericidal antibiotics and that measuring ROS production with the more stable probe, H_2_DCFDA, is reliable (Vinogradov and Grivennikova, 2016; Van Acker and Coenye, 2017). Other studies have also shown that the presence of antioxidants greatly reduces bacteria susceptibility to bactericidal antibiotics (Kohanski et al., 2007; Van Acker et al., 2016). Our findings further support a central role for ROS production by showing a correlation between the ability to generate ROS and the bactericidal activity of TO against SCVs or of the TO-GEN combination against prototypical strains of *S. aureus*. ROS are generated in TO-susceptible SCV strains exposed to TO but are not produced in TO-resistant strains. Furthermore, this ROS production is abolished by the presence of the antioxidant cysteine, concomitant to a loss of antibiotic activity (higher MICs in presence of cysteine). Similarly, we showed that only the synergistic TO-GEN combination was able to produce ROS in prototypical *S. aureus* strains when compared to that seen with either TO or GEN alone. It would have been interesting to place our fluorescence plate reader under anaerobic conditions to measure ROS and to see if induction of ROS by TO or TO-GEN is attenuated in such conditions. Finally, in time-kill experiments performed with and without oxygen, we observed that the bactericidal activity of the TO-GEN combination against WT *S. aureus* was higher when grown aerobically (Figure 6B), whereas TO could efficiently kill the genetically stable SCV *hemB* mutant in both aerobic and anaerobic conditions (Figure 7). Noteworthy, one needs to keep in mind that respiratory deficient SCVs that arise from a point mutation in the *hemB* gene may not be entirely equivalent to the small colony phenotype that arises from fermentative growth of prototypical strains under anaerobic conditions. Indeed, although in many ways similar metabolically, compensatory responses to deal with lactic acid or NADH accumulation due to the loss of respiration in SCVs (Proctor, 2019) may differ between the two types of small colony phenotypes. Whether or not this improves the killing of SCVs by TO remains to be further investigated.

Under a selective pressure *in vitro*, the emergence of resistance to TO in *S. aureus* involves mutations in *atpE* in the SCV background and mutations in *ndh2* and *qoxC* for resistance of the WT strain to the TO-GEN combination. However, these adaptations come at a cost. Indeed, the reduced ATP production for NewbouldΔ*hemB*_*ccpA*_*atpE* translates into a lower ability to colonize tissues *in vivo*. Likewise, ATCC 29213_*ndh2*_*qoxC* does not infect tissue as well as its parent counterpart. While its fitness alteration is not as important as seen with the *atpE* mutant, its resistance to the TO-GEN combination is also minimal. Noteworthy, neither an *atpE*, *ndh2* or *qoxC* mutation was ever found in clinical SCVs (Proctor, 2019).

Other extensively studied ATP synthase subunit c inhibitors may provide additional insights on the mechanism of TO action. For example, the mycobacterial-specific ATP synthase inhibitor, bedaquiline, can also act as a H + /K + antiporter and can gradually acidifies the cytoplasm (Hards et al., 2018). Besides, an interesting observation from our study was the increase in the uptake of GEN (∼1.6-fold) in prototypic *S. aureus* in presence of TO. From this observation, we hypothesize that proton extrusion from respiration is not as much dissipated when the ATP synthase activity is inhibited by TO, i.e., a membrane energization that is favorable to aminoglycoside uptake. Such a result and interpretation is similar to that previously reported by Miller et al. (1980) who demonstrated that the universal ATP synthase inhibitor *N*,*N*’-dicyclohexyl carbodiimide enhances aminoglycoside uptake. Similarly, a positive impact on the proton motive force is seen in ATP synthase mutants of *S. aureus* (Vestergaard et al., 2016) or *E. coli* (Jensen and Michelsen, 1992; Lobritz et al., 2015), enhancing gentamicin uptake and ROS production (Brynildsen et al., 2013). Hence, as seen in the present study, the combination of an ATP synthase inhibitor and an aminoglycoside results in an increase susceptibility to this class of antibiotics.

## Conclusion

A reduction in membrane potential is observed in the presence of TO for both the prototypic and SCV phenotypes of *S. aureus*. The membrane potential of SCV strains apparently drops below a critical level and ROS are only produced in such TO-susceptible SCV strains. Combining TO and GEN increases the membrane potential of prototypic *S. aureus* and the combination greatly increases ROS production. ROS production and increase in GEN cell uptake in the presence of TO may explain the synergy between TO and GEN against prototypic *S. aureus*.

## Data Availability Statement

All datasets generated for this study are included in the article/Supplementary Material.

## Ethics Statement

The animal experiments were carried out according to the guidelines of the Canadian Council on Animal Care and the institutional ethics committee on animal experimentation of the Faculté des Sciences of Université de Sherbrooke, which specifically approved the protocol used for this study.

## Author Contributions

J-PL did all the experiments to collect data on antibiotic profiles, membrane potential, reactive oxygen species production and kill kinetics. GM performed all *in vivo* experiments with animals. IG generated the resistant mutants and prepared strains for whole-genome sequencing. AD-D established the experimental conditions for determination of cell surface hydrophobicity. SC articulated data presentation. P-ÉJ and SR prepared the strategy for sequencing and bioinformatics and IG, FM, P-ÉJ, and SR performed the analyses. KB, ÉM, and FM established the research plan and obtained funding. J-PL and GM produced the initial draft of the manuscript. All authors contributed to data interpretation and generated the final version of the manuscript. FM is the corresponding author.

## Conflict of Interest

The authors declare that the research was conducted in the absence of any commercial or financial relationships that could be construed as a potential conflict of interest.

## Ackowledgments

We are grateful to Calcul Québec, Compute Canada and the Centre de Calcul Scientifique of the Université de Sherbrooke for access and technical support while using the Mammouth-mp2 supercomputer.

## Supplementary Material

FIGURE S1 Stepwise increase in gentamicin (GEN) MIC for the isolate population resulting from serial passage in broth containing a sub-minimal inhibitory concentration of the tomatidine (TO)-GEN combination. The TO concentration was maintained at 8 μg/ml. At the indicated passage (colored bars), the isolate numbers of the sequenced strains that were retained for further investigation are provided. The GEN MIC in the combination is also indicated for the specific and purified isolates that were sequenced. The isolate numbers are traceable in the sequencing data (Supplementary Table S1).

FIGURE S2 Cell surface hydrophobicity of additional *S. aureus* strains. High and low cell surface hydrophobicity are characteristics that applied to SCVs (Δ*hemB* strains) and prototypical strains, respectively.

FIGURE S3 Membrane potential (MFI) of *S. aureus* strains in the presence of tomatidine. The MFI was relative to that of each strain grown without antibiotic (control).

FIGURE S4 Mouse thigh infections by *S. aureus* strains. NewbouldΔ*hemB* colonization was compared to that of NewbouldΔ*hemB*_*ccpA*. CFU were determined 8 h post-infection. Each symbol represents one thigh tissue. The median for each group is indicated by the horizontal bar. Significance between the median log_10_ CFU per gram of tissue obtained for each strain was determined using an unpaired t-test two tailed; ns, not statistically significant.

TABLE S1 Mutations associated with an increased resistance to either GEN or TO-GEN.
