# Supplementary figures and images for: Bactericidal Activity of the Bacterial ATP Synthase Inhibitor Tomatidine and the Combination of Tomatidine and Aminoglycoside Against Persistent and Virulent Forms of Staphylococcus aureus
Source: Front Microbiol. 2020 May 5;11:805. doi: 10.3389/fmicb.2020.00805 (PMC7216300; doi:10.3389/fmicb.2020.00805)

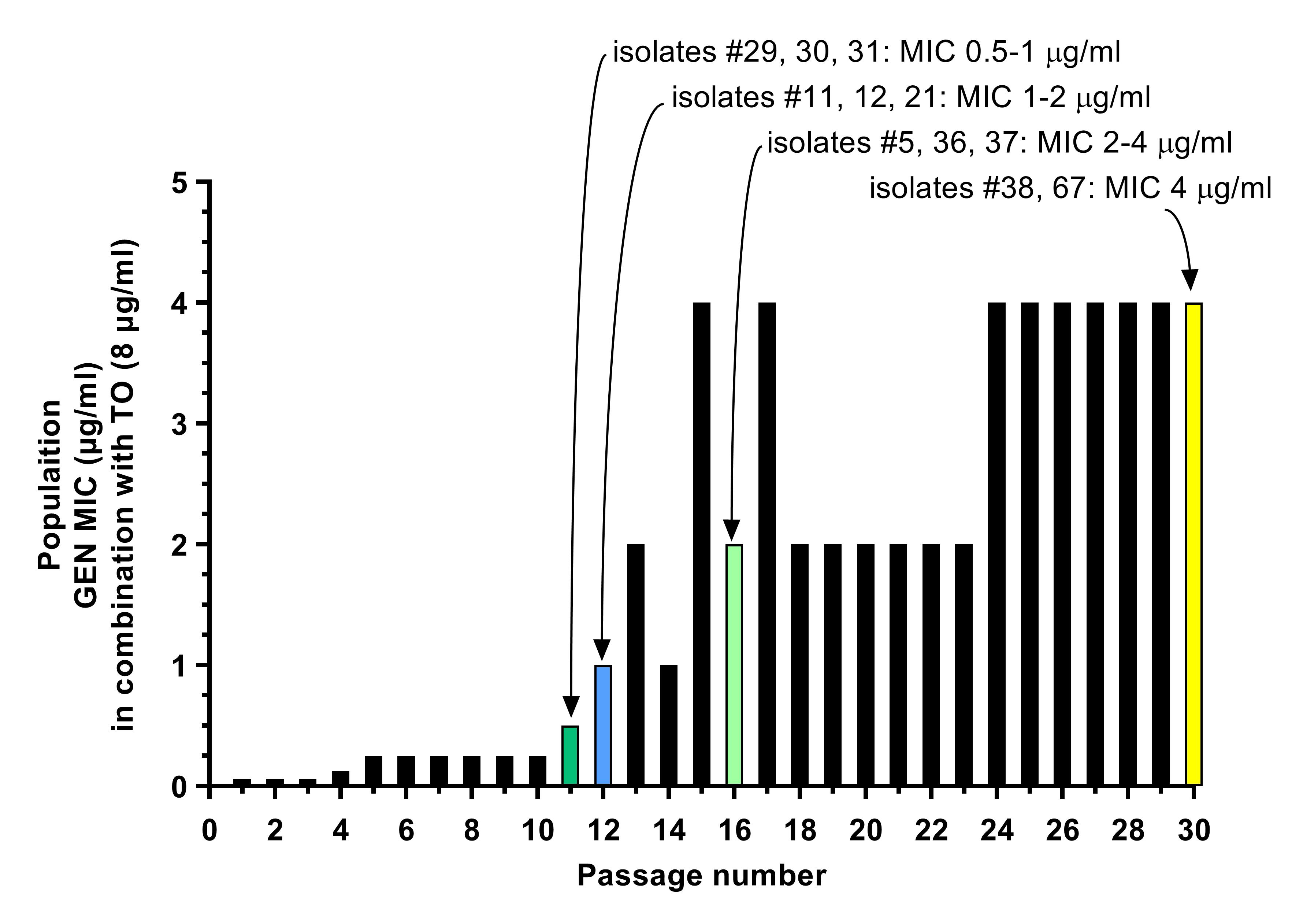

Supplement: FIGURE S1 — Stepwise increase in gentamicin (GEN) MIC for the isolate population resulting from serial passage in broth containing a sub-minimal inhibitory concentration of the tomatidine (TO)-GEN combination. The TO concentration was maintained at 8 μg/ml. At the indicated passage (colored bars), the isolate numbers of the sequenced strains that were retained for further investigation are provided. The GEN MIC in the combination is also indicated for the specific and purified isolates that were sequenced. The isolate numbers are traceable in the sequencing data (Supplementary Table S1). [file Image_1.JPEG]

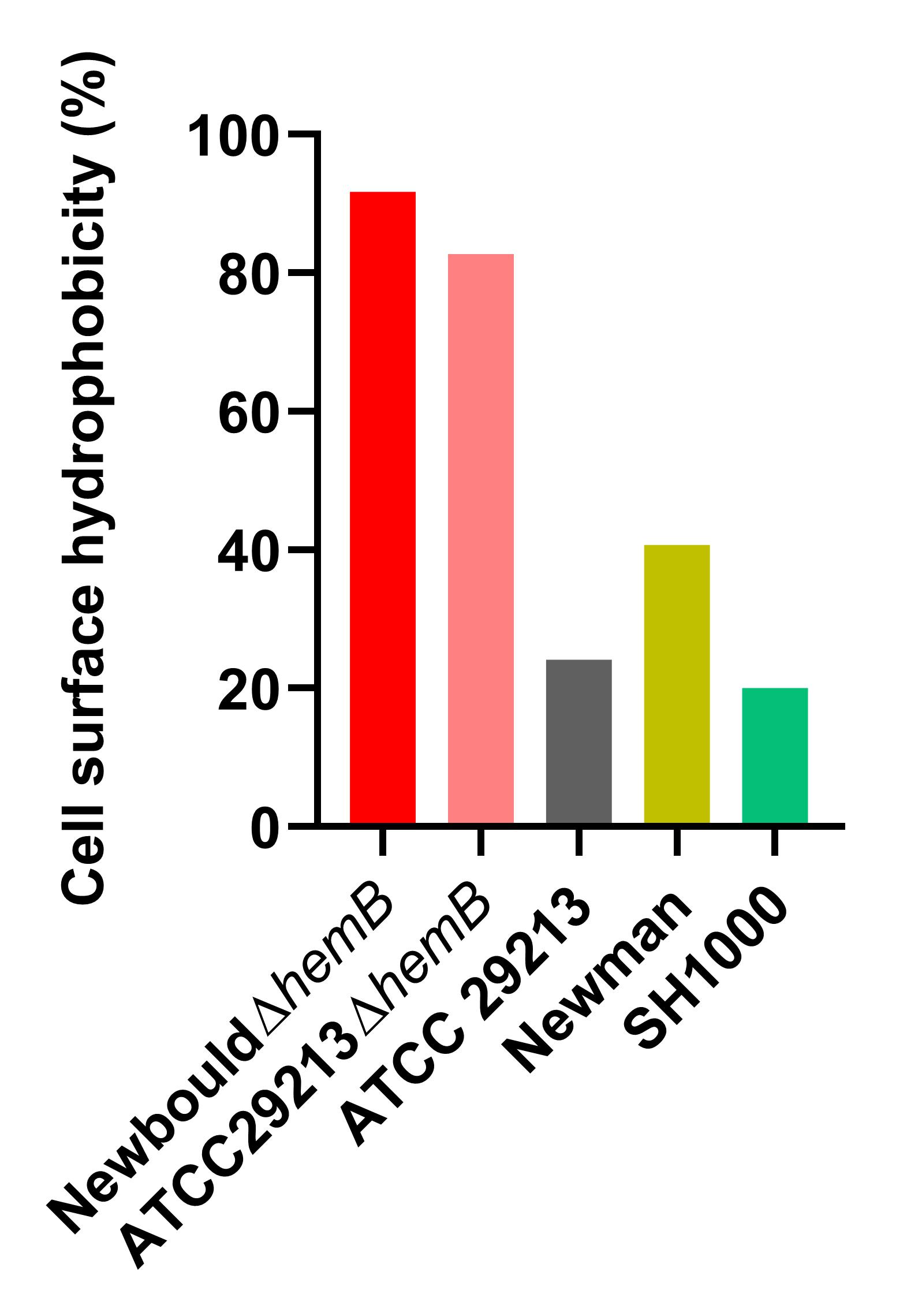

Supplement: FIGURE S2 — Cell surface hydrophobicity of additional S. aureus strains. High and low cell surface hydrophobicity are characteristics that applied to SCVs (ΔhemB strains) and prototypical strains, respectively. [file Image_2.JPEG]

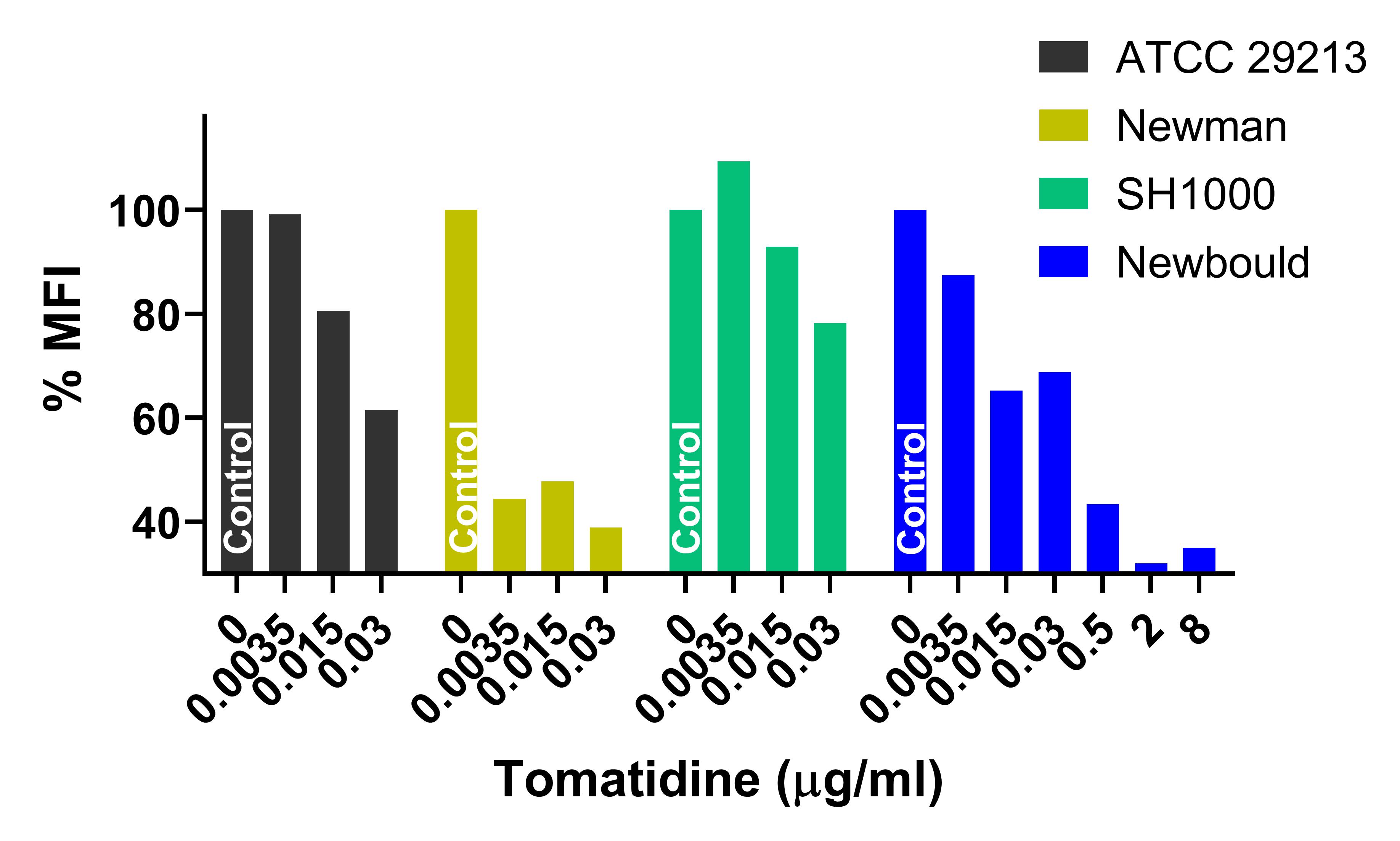

Supplement: FIGURE S3 — Membrane potential (MFI) of S. aureus strains in the presence of tomatidine. The MFI was relative to that of each strain grown without antibiotic (control). [file Image_3.JPEG]

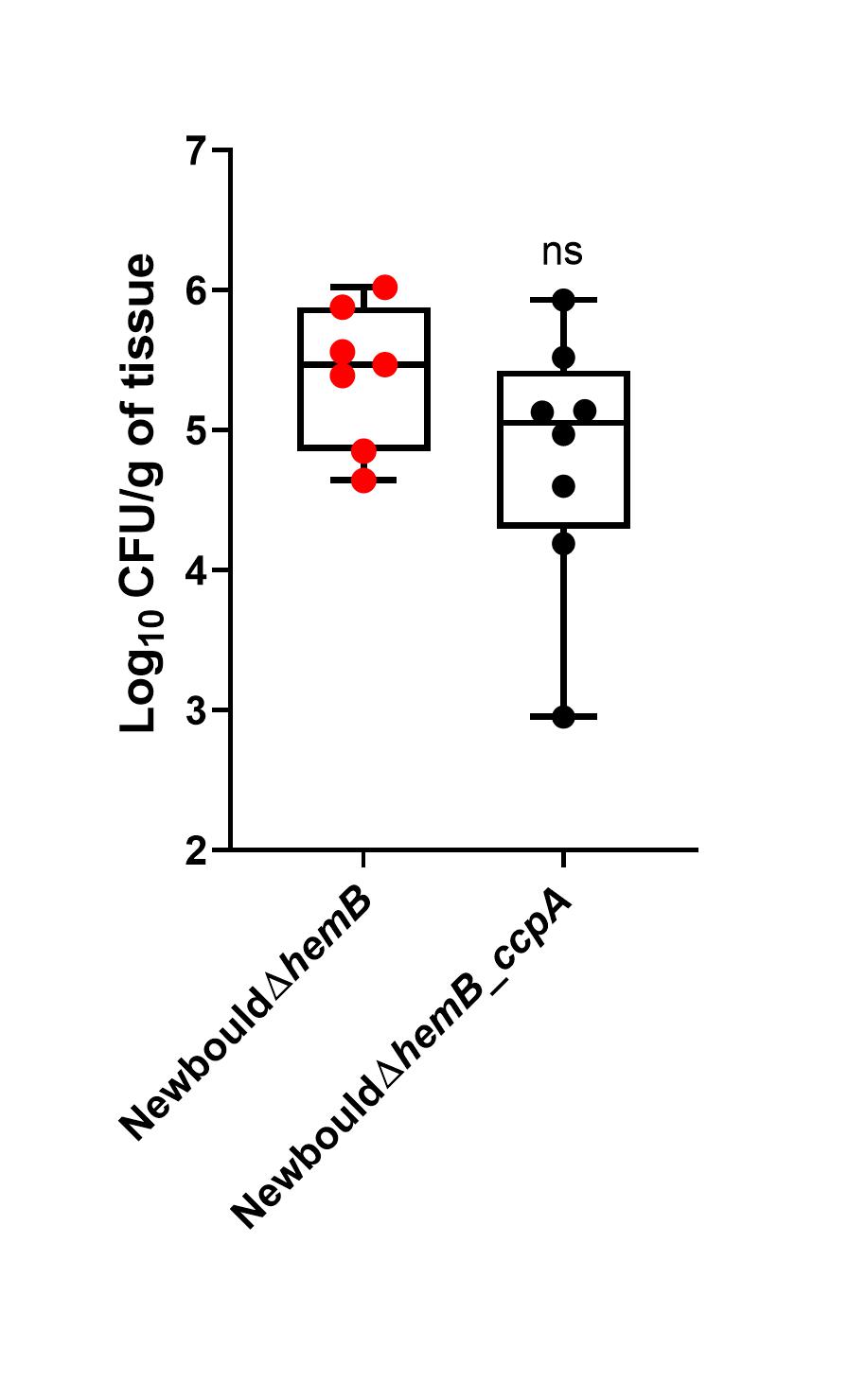

Supplement: FIGURE S4 — Mouse thigh infections by S. aureus strains. NewbouldΔhemB colonization was compared to that of NewbouldΔhemB_ccpA. CFU were determined 8 h post-infection. Each symbol represents one thigh tissue. The median for each group is indicated by the horizontal bar. Significance between the median log10 CFU per gram of tissue obtained for each strain was determined using an unpaired t-test two tailed; ns, not statistically significant. [file Image_4.JPEG]
